# Characterization of Composite Powder Feedstock from Powder Bed Fusion Additive Manufacturing Perspective

**DOI:** 10.3390/ma12223673

**Published:** 2019-11-07

**Authors:** Eskandar Fereiduni, Ali Ghasemi, Mohamed Elbestawi

**Affiliations:** Department of Mechanical Engineering, McMaster University, Hamilton, ON L8S 4L7, Canada; ghasemia@mcmaster.ca

**Keywords:** additive manufacturing, powder bed fusion, selective laser melting, regular mixing, ball milling, flowability, Ti-6Al-4V

## Abstract

This research aims at evaluating the characteristics of the 5 wt.% B_4_C/Ti-6Al-4V composite powder feedstock prepared by two different categories of mechanical mixing for powder bed fusion (PBF) additive manufacturing (AM) of metal matrix composites (MMCs). Microstructural features, particle size, size distribution, sphericity, conditioned bulk density and flow behavior of the developed powders were examined. The flowability of the regularly mixed powders was significantly lower than that of the Ti-6Al-4V powder. However, the flowability of the ball-milled systems was a significant function of the milling time. The decrease in the flowability of the 2 h ball-milled powder compared to the Ti-6Al-4V powder was attributed to the mechanical interlocking and the entangling caused by the B_4_C particles fully decorating the Ti-6Al-4V particles. Although the flattened/irregular shape of powder particles in the 6 h milled system acted to reduce the flowability, the overall surface area reduction led to higher flowability than that for the 2 h milling case. Regardless of the mixing method, incorporation of B_4_C particles into the system decreased the apparent density of the Ti-6Al-4V powder. The composite powder obtained by 2 h of ball milling was suggested as the best possible condition, meeting the requirements of PBF–AM processes.

## 1. Introduction

Metal matrix composites (MMCs) are outstanding materials bilaterally benefitting from the properties of at least two constituents: the metal matrix (usually an alloy), and the reinforcement (in general, an oxide, an intermetallic compound, a carbide or a nitride) [1,2,3]. Incorporation of reinforcements into the metallic matrix is generally associated with the improvement in hardness, specific strength, wear resistance, fracture toughness, and stiffness compared to the monolithic counterparts [4,5,6,7]. Owing to their desired structural and functional properties, MMCs have found their application in numerous technological fields including automotive, aerospace and biomedical industries [8,9].

Several conventional manufacturing processes already exist for incorporating reinforcements into the metallic matrices to produce a wide variety of MMCs [10,11,12,13,14]. However, it is rather challenging to fabricate geometrically complex parts using these processes. Additive manufacturing (AM) is regarded as a major revolution in the manufacturing technology and competes with conventional manufacturing processes in many aspects, including, but not limited to, the design freedom, fabrication cost and time, accuracy and the part quality [15,16].

Powder bed fusion (PBF) refers to the AM processes in which an object is manufactured layer-by-layer from a batch of loose powder using a mobile heat source. During this process, a thin layer of powder is deposited on the building platform by the recoater. The powder flow behavior during the recoating process plays a critical role on the uniformity, surface roughness and the thickness of the deposited powder layer and consequently, the dimensional accuracy of the final part [17,18]. On the other hand, the powder bed packing density directly affects the density and mechanical properties of the additively manufactured components [19,20]. Therefore, the flowability and the packing density of the powders need to be investigated prior to the AM process to ensure the soundness of the final parts [21,22,23,24].

The desired properties of MMCs are achieved when the reinforcements are homogeneously distributed throughout the matrix with a strong reinforcement/matrix interfacial bonding [25,26]. Conventional MMC fabrication methods generally yield inhomogeneous microstructures, making it rather difficult to fully exploit the strengthening potentials of reinforcements. Therefore, it is of crucial importance to develop new fabrication routes providing a more homogenous distribution of reinforcements within the matrix [16]. The noticeably localized melt pool and the extremely high solidification rates associated with the PBF–AM technology can lead to MMC structures which are much more homogenous than the conventionally processed parts [27]. However, there are still some challenges with the PBF–AM processing of MMCs for achieving highly uniform microstructures due to the following reasons [3,28]:

- In nano-composites, reinforcing particles tend to agglomerate and form coarsened clusters in the matrix due to the presence of van der Waals attraction forces among them.
- A large difference between the densities of the reinforcing particles and the liquid matrix encourages the non-uniform distribution of reinforcements in the microstructure.
- The convection flows (i.e., Marangoni effect) induced in the melt pool may not be sufficient to disperse the reinforcing particles throughout the system.

Therefore, when it comes to the PBF–AM of MMCs, particular emphasis should be placed on pre-processing of the composite powder feedstock in order to achieve parts with homogenous microstructures and consequently, uniform mechanical properties. Development of a suitable composite powder feedstock with a uniform distribution of reinforcing particles is the first step to mitigate the mentioned challenges.

Since the feedstock of MMCs is not commercially available for AM processing [29], several techniques have been employed in recent years to prepare these powders. The mechanical routes such as regular mixing [30,31,32] and ball milling [33,34,35,36,37,38,39], and non-mechanical methods including gas atomization of a pre-alloyed system [40,41], agent-assisted deposition [42,43] and electrodeposition [44,45] are among these methods. Compared to the mechanical mixing routes, which have attracted a great deal of attention in recent years, the non-mechanical methods have been rarely adopted to prepare composite powder feedstocks for AM purposes. Although the non-mechanical methods can probably produce composite powder systems with a better flowability and a more uniform distribution state of powder constituents compared to the mechanical routes, they are much more complex and expensive. Due to their low cost as well as their applicability to many powder systems, the mechanical routes are the most frequently used methods in powder feedstock preparation for the PBF–AM of MMCs [29,46].

Incorporation of the guest powder particles (e.g., ceramic particles) into the host powder particles (usually metallic particles) leads to the production of composite powder feedstocks with different characteristics from the individual constituents. The particle size, particle size distribution, and distribution state of the guest powder particles are among these features which dictate the apparent density, flowability, and laser absorptivity of the composite powder [15,16]. The laser absorptivity influences the heat absorption, and the melt pool size [44,47,48], while the flowability and the apparent packing density of the powder play crucial roles in layer thickness (dimensional accuracy) and density of the final part [17,49,50,51,52]. A literature review of the AM processing of MMCs reveals that the mixing of powders, in most cases, has been performed to achieve a distribution of free (non-attached) guest particles throughout the mixed powder system [30,31,32,33,34,35,36,37,38,39,53,54,55]. On the other hand, preserving the spherical shape of metallic powder particles has been considered in some of these studies [36,53,55,56]. However, the effect of powder preparation routes and process variables on the composite powder features affecting the quality of AM processed parts is still unclear and needs in-depth analysis and characterization from the AM perspective to obtain high-quality MMCs.

The present research aims to study the characteristics of the mechanically mixed 5 wt.% B_4_C/Ti-6Al-4V (Ti64) composite powder feedstock from the PBF–AM viewpoint. For this purpose, the regular mixing and the ball-milling methods were employed with different mixing times to prepare a wide variety of mixed powder systems. The effects of the mixing method and the mixing time on the size, size distribution, sphericity, shape, distribution state of guest particles, phase formation, plastic deformation, apparent density, and flow behavior of the prepared composite powder systems were studied. Moreover, the mechanisms involved in the flow behavior of the developed feedstocks were identified, and the best possible powder feedstock meeting the requirements of PBF–AM processing of MMCs was proposed.

## 2. Materials and Methods

### 2.1. Powder Preparation

**Figure 1:**
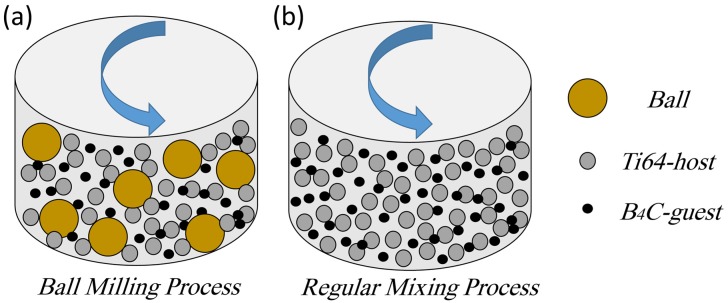
Schematic illustration of: (**a**) ball milling and (**b**) regular mixing processes.

The powders used in this research were Ti64 (15–45 µm) and B_4_C (1–3 µm) named as “host” and “guest” powders, respectively. The nominal chemical composition of these powders is provided in Table 1. For preparing the composite powder feedstock, the regular mixing and the ball-milling processes were employed, which are schematically shown in Figure 1. In both cases, the mixed powder systems contained 5 wt.% B_4_C (guest), and the powders were mixed under a protective argon gas atmosphere to avoid oxidation. The mixing process was performed using a high-performance planetary Pulverisette 6 machine at a fixed rotational speed of 200 rpm and mixing times in the range of 1 to 6 h. The regularly mixed and the ball-milled samples have been marked as R(1–6) and B(1–6), respectively, depending on their mixing time. In the regular mixing, the powders were mixed without balls. However, stainless steel balls with a diameter of 10 mm were added to the system in the ball-milling process. The ball-to-powder ratio was selected to be 5:1, and every 30 min of milling was followed by a 15 min pause in order to avoid a temperature increase during the process [57]. For each composite powder system, the mass was measured after the mixing to find the material loss.

### 2.2. Powder Characterization

#### 2.2.1. Microstructure and XRD Analysis

The morphology of the starting Ti64 (host) and B_4_C (guest) powders, as well as the composite powder systems, were studied using Vega Tescan scanning electron microscopy (SEM) operating at an accelerating voltage of 20 kV. The X-ray diffraction (XRD) analysis was employed to study the effect of the mixing method and the mixing time on the phase formation and the plastic deformation of the developed composite powder feedstocks. This analysis was performed at ambient temperature over a wide range of 2θ= 20°–80° using a PANalytical X’Pert powder X-ray diffractometer (Westborough, MA, USA, Cu Kα target, operating at the voltage and the current of 45 kV and 35 mA, respectively, with a step size of 0.0167°) equipped with an X-ray monochromator. In order to have a better understanding of the microstructural features, the starting Ti64 and composite powders were also sectioned and characterized using SEM.

#### 2.2.2. Particle Size, Size Distribution and Sphericity

A Retsch Camsizer X2 machine (Haan, Germany) was used to measure the particle size, size distribution and sphericity of the Ti64 and the developed 5wt.% B_4_C/Ti64 composite powder systems fabricated through the regular mixing and ball-milling methods. This equipment utilizes a high-resolution dual-camera system to characterize fine and agglomerating powders ranging from 800 nm to 8 mm in diameter [58]. The reported results are the average of three measurements. The sphericity of the powder particles was calculated based on the equation suggested by the ISO 9276-6 standard [59]:

(1) $$Sphericity=\frac{4\pi A}{P^{2}}$$

where P and A are the measured circumference and area covered by a particle projection, respectively. Given the fact that the sphericity of an ideal sphere is unity, deviation from the ideal spherical shape results in lower sphericities.

#### 2.2.3. Flow Characteristics

The flow behavior of powder particles could be characterized using different techniques such as ring shear cell tester, Hausner ratio (HR), angle of repose (AOR)/Hall flowmeter, avalanche angle and Freeman Technology 4 (FT4) powder rheometer [60,61,62]. When deciding to choose one of these techniques, characteristics of both the technique and the process need to be considered since the selected flowability measurement technique should be as close to the employed process as possible. In recent years, the FT4 Powder Rheometer has emerged as a unique technology to measure the flow behavior of the powder, whilst the powder is in motion. In this device, a precision ‘blade’ is rotated and moved downwards through a fixed mass of the powder bed to establish a flow pattern. The work required to drive the rotating impeller a certain distance into the powder bed yields the flow energy. Due to its dynamic nature, this technique is capable of differentiating the flowability of powders that exhibit similar behavior under other flow measurement techniques [63]. The FT4 powder rheometer technique provides measurement of several parameters related to the process performance of powders. The interaction of the precision blade with the powder in this technique resembles that of recoater/powder in the PBF–AM process. It is worth noting that the control over the speed of the blade provided by this technique enables the characterization of powder sensitivity to the changes in flow rate. This unique feature also facilitates analysis of the powder flowability for different PBF–AM machines with different recoater speeds.

The flow characteristics of the host powder, as well as the developed composite powder feedstocks in the present study, were measured using an FT4 powder rheometer (Freeman technology, Tewkesbury, UK). In order to study the powder rheological properties using this technique, the standard “Stability and Variable Flow Rate (SVFR)” method was employed, which consists of a stable tip speed zone with seven test cycles followed by a variable tip speed zone having four test cycles. During each test cycle, the precision blade rotated downwards and upwards through the fixed mass of powder to establish a flow pattern, where the powder resistance to the blade yielded the flow properties. During the stability part, the blade operated with a tip speed of −100 mm/s (anti-clockwise) and a helix angle of 5°. For the variable flow rate zone, the tip speed varied as −100, −70, −40 and −10 mm/s for the test cycles eight, nine, ten and eleven, respectively, with the same helix angle of 5°. The upward speed remained constant at 40 mm/s, and the helix angle was −5° upwards throughout the experiment. It is noteworthy that before all tests, a conditioning cycle was performed that involves the downward and then upward movement of the blade into the powder bed to gently slice the powder and provide a reproducible, uniform and low-stress packing state, allowing an objective comparison of samples. For each sample in this study, three tests were run to ensure consistency of the results. The flow characteristics of the samples were studied by analyzing the variation in the flow energy as well as by measuring the basic flowability and specific energy (SE). In addition, the conditioned bulk density (CBD) of the samples was examined to study the powder bed density.

The basic flowability, defined as the ability of the powder to flow when forced, is qualitatively measured as the basic flow energy (BFE). The BFE represents the energy required for the rotation of the blade for the seventh test cycle ($\mathrm{BFE}=E_{\mathrm{test}\;7,\;\mathrm{down}}$) of the stable tip speed part.

The SE shows the energy required to establish a particular flow pattern in a precise volume of conditioned powder and is defined as the average energy of the upward blade rotation for the 7th and 8th test cycles divided by the mass of the remaining powder in the vessel (Equation (2)). By gently lifting the powder, the upward motion of the blade generates a low stress and unconfined flow mode in the powder.

(2) $$\mathrm{SE}=\frac{\frac{E_{\mathrm{test}\;7,\mathrm{up}}+E_{\mathrm{test}\;8,\mathrm{up}}}{2}}{m_{\mathrm{split}}}$$

where $m_{\mathrm{split}}$ is the mass of the powder after the excess powder is removed.

The CBD describes the packing state or the density of the powder in its reference state. In order to measure the CBD, each powder system was gently filled in a 25 mL volume splitting cylindrical vessel with a diameter of 25 mm. The conditioning process was performed with a conditioning blade, which slices the powder bed to remove the excess air and create a uniform powder bed with a low-stress packing state. After conditioning, the vessel was split in order to remove the excess mass of powder, so that the remaining powder had a volume of 25 mL. For each sample, three measurements were performed, and the average value was reported as the CBD, based on Equation (3):

(3) CBD=msplitvsplit

In which $v_{\mathrm{split}}$ signifies the volume of the powder (the vessel volume), after the excess powder is removed.

## 3. Results and Discussions

### 3.1. XRD Analysis: Plastic Deformation and Phase Formation

**Figure 2:**
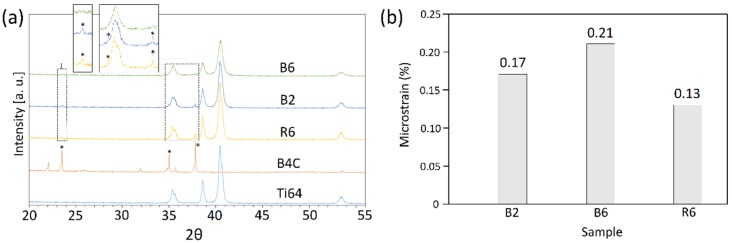
(**a**) XRD patterns of starting Ti-6Al-4V (Ti64) and B_4_C powders, as well as composite powder systems prepared by regular mixing and ball milling, and (**b**) The microstrain of Ti64 constituent in the developed composite powder systems derived from the XRD patterns.

Figure 2a presents the XRD patterns of the starting host and guest powders, as well as the developed composite powder systems subjected to the regular mixing and ball milling for different mixing times. The diffraction peaks of Ti64 in the R6 system had almost the same position and intensity as that of the starting Ti64. However, those for the B2 and the B6 systems exhibited the Ti64 diffraction peaks with a decreased intensity and increased width. The observed phenomenon was more pronounced for the B6 sample than for the B2 system. Moreover, a close examination of the Ti64 peaks in the ball-milled systems revealed that the severe plastic deformation induced in the ball milling process led to the shift in the peaks’ position due to the structural changes such as the crystallite refinement and the accumulation of micro-strain [64,65]. The lattice micro-strain of the Ti64 constituent in the composite powder systems was determined using the standard Williamson–Hall analysis as follows [66]:

(4) $$\beta \cos\theta =\left[\frac{k\lambda }{t}\right]+4\varepsilon \sin\theta$$

where k is the shape factor (0.9), λ represents the wavelength of the X-ray (1.5406), θ signifies the diffraction angle, t is the effective crystallite size, β is the full width at the half maximum of the XRD peak, and ε is the micro-strain. By constructing a linear plot of (βcosθ) against (4sinθ), the slope gives the strain (ε). According to the micro-strain results provided in Figure 2b, the B2 and B6 systems showed increased lattice strain compared to the regularly mixed feedstocks. Also, longer milling times led to higher lattice strain (B6 compared to B2) due to the higher levels of plastic deformation imparted to the powder particles.

Referring to Figure 2a, all the peaks obtained for the composite powder feedstocks corresponded to those for the Ti64 and B_4_C powders due to two probable scenarios: (i) no in-situ reaction has been activated in the system during the applied range of mixing times, or (ii) if formed, the amount of in-situ synthesized phases is below the detection limit of the XRD analysis. As indicated in Figure 2a, the intensity of the B_4_C peaks in the R6 composite powder system decreased compared to those for the starting B_4_C powder. When employing the ball-milling method, the intensity of these weak peaks further decreased for the B2 sample and finally, disappeared in the B6 system.

### 3.2. Microstructural Characterization

**Figure 3:**
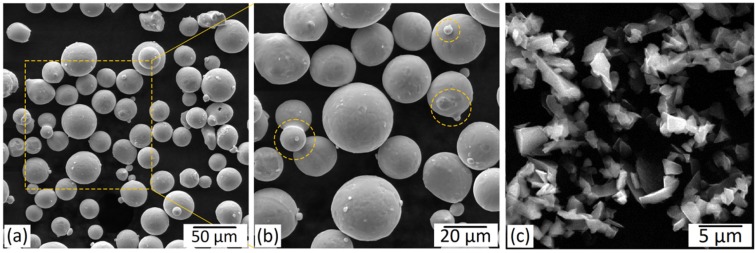
SEM micrographs of starting: (**a**,**b**) Ti-6Al-4V (host) and (**c**) B_4_C (guest) powder particles. (**b**) Higher-magnification micrographs of the selected region in (**a**). The circles in (**b**) indicate the satellites.

Figure 3 presents SEM micrographs of the starting host Ti64 and the guest B_4_C powders used in this research. As observed in Figure 3a,b, the starting host powder particles had an almost spherical morphology and a very smooth surface, which are both characteristics of the gas-atomized Ti64 powders [17]. However, B_4_C particles exhibited an irregular morphology (Figure 3c). Despite their smooth surface, some of the Ti64 particles have satellites. These satellites are formed when the finer solidified particles stick to the molten or semi-molten surface of the coarser ones as a result of the in-flight collisions before the solidification of the coarser molten droplets [67].

**Figure 4:**
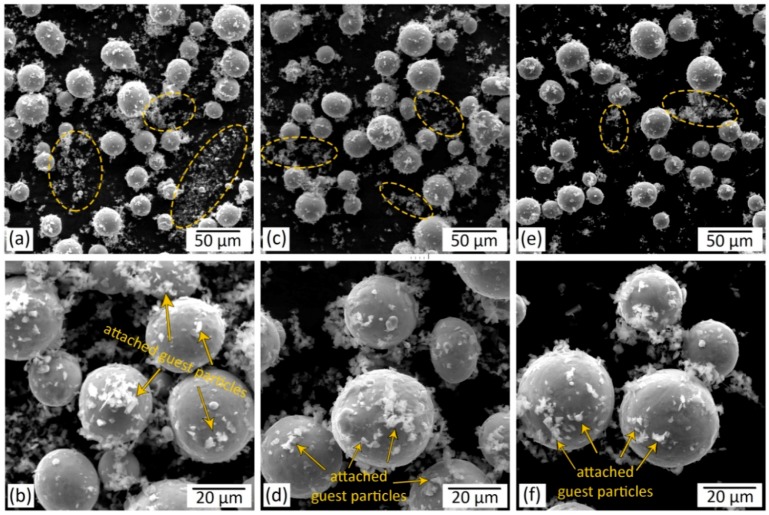
SEM micrographs of 5 wt.% B_4_C/Ti-6Al-4V mixed powder systems subjected to regular mixing for: (**a**,**b**) 1 h (R1); (**c**,**d**) 3 h (R3); and (**e**,**f**) 6 h (R6). The ellipses in (**a**,**c**,**e**) indicate the free B_4_C (guest) particles that are not attached to the Ti-6Al-4V (host) particles and are agglomerated.

**Figure 5:**
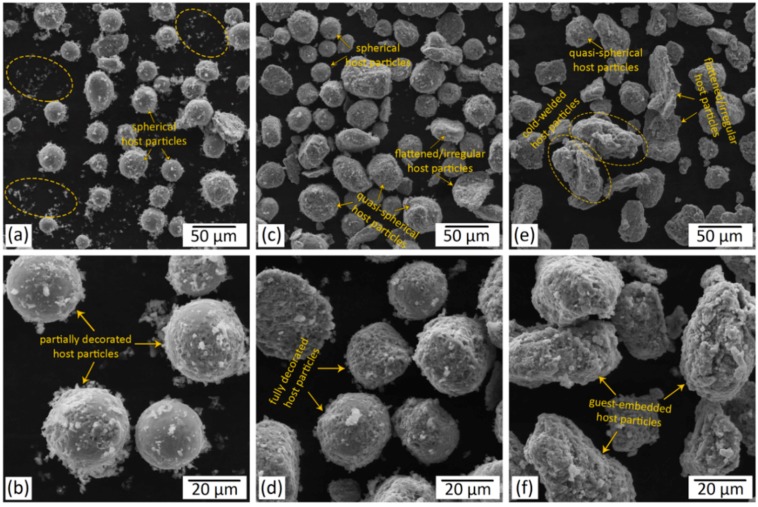
SEM micrographs of 5 wt.% B_4_C/Ti-6Al-4V mixed powder systems subjected to ball milling for: (**a**,**b**) 1 h (B1); (**c**,**d**) 3 h (B3); and (**e**,**f**) 6 h (B6). The ellipses in (**a**) show the free B_4_C (guest) particles that are not attached to the Ti-6Al-4V (host) particles.

The micrographs of the regularly mixed and ball-milled composite powders subjected to various mixing times are provided in Figure 4 and Figure 5, respectively.

**Figure 6:**
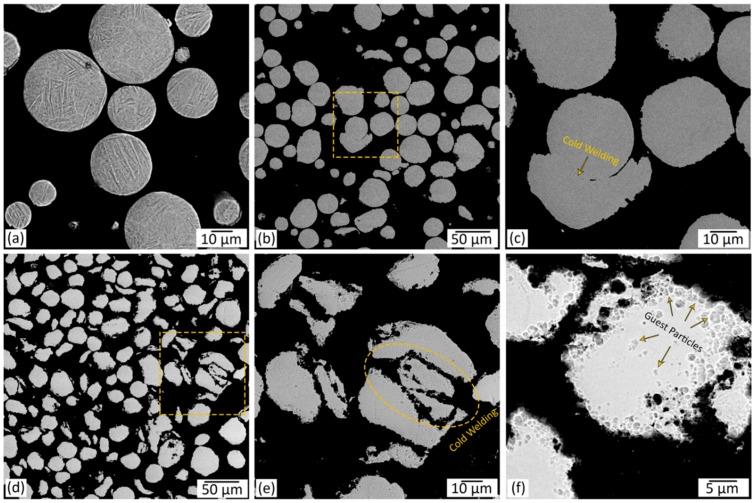
Cross-sectional SEM micrographs of: starting Ti-6Al-4V powder showing a martensitic microstructure after etching (**a**); 5 wt.% B_4_C/Ti-6Al-4V system subjected to ball milling for: (**b**,**c**) 2 and (**d**–**f**) 6 h. (**c**,**e**) are higher magnification micrographs of the regions shown in (**b**,**d**), respectively.

The cross-sectional SEM micrographs of the starting Ti64 powder, as well as the B2 and the B6 powder systems, are presented in Figure 6.

As can be observed in Figure 4, the Ti64 host particles maintained their spherical morphology when employing the regular mixing method even with mixing times as long as 6 h (R6). However, even after a long mixing time of 6 h (R6), many of the guest particles were not attached to the host powder particles (Figure 4e), meaning that noticeably longer mixing times may still be required to provide more guest-to-host attachment. The guest particles that are not attached to the host powder particles tend to form agglomerates (as depicted in Figure 4a,c,e). Despite this fact, the regular mixing method has been adopted in numerous studies to produce composite powders due to its relative simplicity [30,31,32,68,69].

Depending on the employed mixing time, the host powder particles experienced different levels of plastic deformation during the ball-milling process (Figure 5 and Figure 6). At a relatively short milling time of 1 to 2 h, the host power particles preserved their spherical shape (Figure 5a and Figure 6b,c). Higher amounts of plastic deformation imparted to the system by a longer milling time of 3 h (B3) resulted in some spherical to quasi-spherical/irregular shape change (Figure 5c). Also, due to the extended time that the hard guest particles were hitting their surface, the enhanced milling time increased the surface roughness of the host powder particles. In the prolonged milling time of 6 h (B6), the desired spherical shape of the host particles altered to a flattened/irregular shape (Figure 5e,f and Figure 6d–f). Microstructural observations also revealed the cold-welding of the ductile host powder particles during the ball milling process. Longer mixing times were found to cause intensified cold-welding in the applied range of ball milling time (Figure 5 and Figure 6). Even at relatively short mixing time of 1 h, a significant guest-to-host attachment was obtained in the ball milling process, while still a few free B_4_C particles could be observed in the composite powder (Figure 5a,b). Increasing the mixing time to 3 h eliminated the non-attached B_4_C particles and led to the host particles fully decorated by the guest ones (Figure 5c,d). Further enhancement of the mixing time to 6 h caused the embedment of the guest particles into the host powder particles (Figure 5e,f and Figure 6d–f).

The observed shape change and the agglomeration of the particles both observed at relatively long milling times are known as the main issues limiting the application of such composite powder systems in PBF–AM processes [29,36]. Sieving has been suggested as one of the strategies that could be employed to tailor the particle size in the powder systems subjected to the extended milling times [70]. While successful with particle size, this technique does not control the particle shape. Therefore, depending on the ductility of the host powder particles, appropriate milling times need to be found for each system to control the final shape of the particles.

### 3.3. Particle Size, Size Distribution and Sphericity

**Figure 7:**
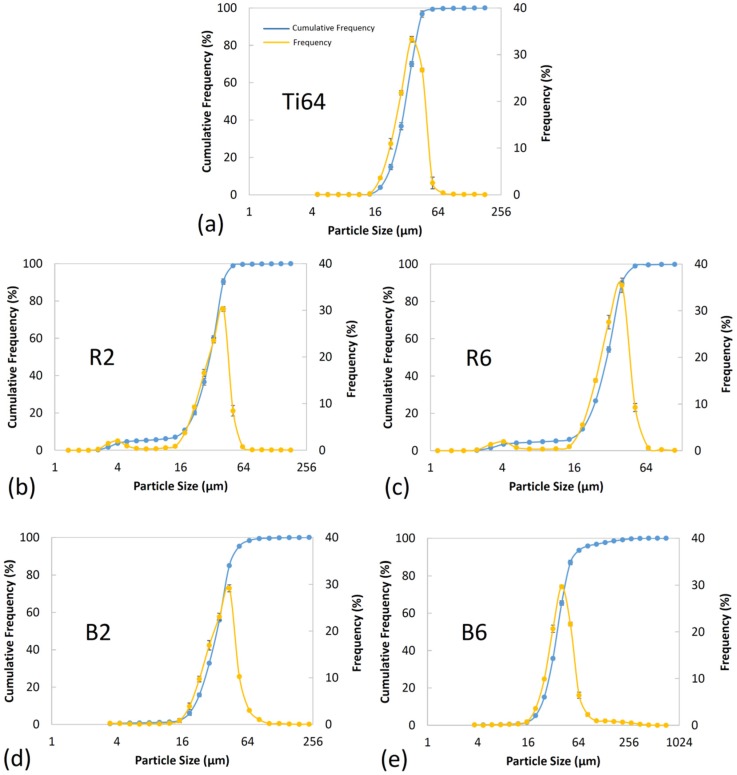
The particle size distribution curves of: (**a**) starting Ti-6Al-4V (Ti64), (**b**) 2 h (R2) and (**c**) 6 h (R6) regularly mixed; (**d**) 2 h (B2) and (**e**) 6 h (B6) ball milled 5wt.% B_4_C/Ti64 composite powder feedstocks.

Figure 7 presents the results of particle size distribution for the starting Ti64 powder as well as the developed 5 wt.% B_4_C/Ti64 powder feedstocks prepared by the regular mixing and ball-milling methods for 2 and 6 h of mixing. As observed in Figure 7b,c, the regularly mixed powder systems have a bimodal size distribution, while the ball-milled composite powders show a mono-modal size distribution (Figure 7d,e). This can be attributed to the non-attached B_4_C particles in the regularly mixed case as opposed to the full attachment of the guest particles to the host powder particles in the ball-milled powder systems. It is worth noting that even a relatively long mixing time of 6 h in the regular mixing method was not successful in full guest-to-host attachment (Figure 4e,f). However, a relatively short mixing time of 1 h in the ball-milling method led to the B_4_C particles being well-attached to the host powder particles (Figure 5a,b). The enhanced milling time provided attachment of more guest particles to the surface of the host particles, eliminating the free guest particles in the composite powder (Figure 5c,d). Application of longer milling times (B6) resulted in the embedment of B_4_C particles into the host powder particles (Figure 5e,f and Figure 6f). The severe weakening (or even disappearance) of the B_4_C diffraction peaks in this system (B6 powder in Figure 2a) may be attributed to their embedment into the ductile host powder particles.

The D10, D50, and D90 of the powder systems derived from the cumulative frequency shown in Figure 7 are provided in Table 2. Referring to Figure 7 and Table 2, the regularly mixed composite powders showed almost the same particle size and particle size distribution as that of the starting Ti64 powder. The slight deviation in D10 and D50 of R2 and R6 systems from those for Ti64 is caused by the presence of free guest B_4_C particles with a significantly smaller particle size compared to the host Ti64 particles (Figure 4). Due to its non-equilibrium nature, the ball-milling process involves persistent deformation, cold-welding, and fracture of powder particles [71,72]. The size of the powder particles subjected to the ball-milling process is determined by the competition between two major mechanisms, namely, cold-welding and fracture [71]. While the cold-welding mechanism facilitates the formation of larger-sized particles by attachment of the host powder particles, the fracture mechanism favors the decrease in particle size. Hence, the refining or coarsening of powder particles during the ball-milling process depends on whether the cold-welding or the fracture mechanism is dominant [73]. For the applied range of milling times, the particle size showed an ascending trend upon increasing the mixing time as a result of the cold-welding mechanism being dominant (Figure 5e and Figure 6b,c,e). For instance, D90 for the B2 and B6 samples is 12% and 35% higher than that of Ti64 powder, respectively. The particle coarsening in the ball-milled composite samples is caused by the guest-to-host attachment as well as the cold-welding of host particles. The decoration of host particles is the dominant factor in increasing the particle size in the B2 sample due to the limited cold-welding caused by the relatively short mixing time (Figure 5 and Figure 6). However, the significant cold-welding induced by the prolonged mixing time in the B6 sample is the main factor governing the particle coarsening, since the guest particles are embedded into the host Ti64 powder (not decorating the host particles). Based on the D90 of the B6 sample (Table 2) and Figure 7, 10% of particles have sizes in the range of 62–275 μm. These particle sizes are noticeably larger than the starting Ti64 powder particles, with 0.3% of particles exceeding 62 μm in size.

**Figure 8:**
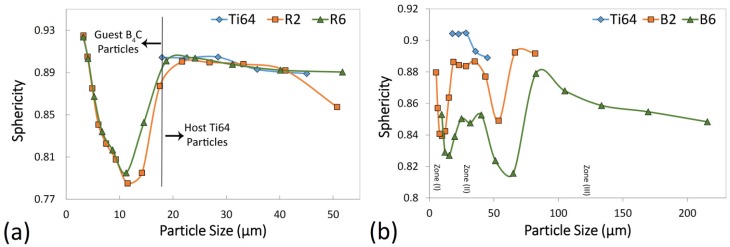
The sphericity of the particles as a function of their size for: (**a**) regularly mixed and (**b**) ball-milled 5 wt.% B_4_C/Ti64 composite powder feedstocks. In each case, the sphericity of the starting Ti-6Al-4V (Ti64) powder is provided as the reference.

Figure 8 shows the sphericity of the powder particles with respect to their size for the starting Ti64 powder, as well as the developed composite powders. In the regular mixing case shown in Figure 8a, the sphericity of the host powder particles is the same as the starting Ti64 particles (18–50 μm). This can be ascribed to the absence of plastic deformation in the regular mixing method as well as the limited attachment of the guest B_4_C particles to the host ones (Figure 4). The non-attached B_4_C particles existing in the R2 and R6 samples showed particle sizes in the range of 3–18 μm, representing the formation of agglomerated guest particles (Figure 4). The relatively low sphericity of these agglomerates originates from their irregular shape (Figure 3c). According to Figure 8b, three different zones can be defined in the sphericity–particle-size curves for the B2 and B6 samples. Zones (I), (II) and (III) refer to the particle sizes in the range of (5–18), (18–65) and (>65) µm for B2, and (10–25), (25–80) and (>80) µm for B6, respectively. Since the B2 and B6 samples are free from non-attached B_4_C particles, Zone (I) signifies the fractured host powder particles showing lower sphericity than the starting Ti64 particles. In Zone (II), the deformation and cold-welding, as well as the decoration of the host particles by the guest ones, lead to the decreased sphericity compared to the Ti64 powder. Since the short milling time of 2 h resulted in the limited cold-welding, the host particles decoration and deformation are believed to be the dominant factors, slightly decreasing the sphericity of the B2 sample. According to Figure 5a,b and Figure 6b,c, most of the host powder particles preserved their spherical shape at short milling times. However, due to the embedment of the guest particles into the host ones in the B6 sample (absence of decoration), deformation and cold-welding of the host particles are responsible for the sphericities lower than the B2 sample. Zone (III) which is mostly visible in the B6 sample represents the cold-welded quasi-spherical/irregular shape powder particles (agglomerates) with sizes much larger than the starting Ti64 particles (Figure 5e,f and Figure 6d–f). The sphericity of these agglomerates follows a decreasing trend by increasing their size.

### 3.4. Flow Behavior and Conditioned Bulk Density (CBD)

#### 3.4.1. Flowability

**Figure 9:**
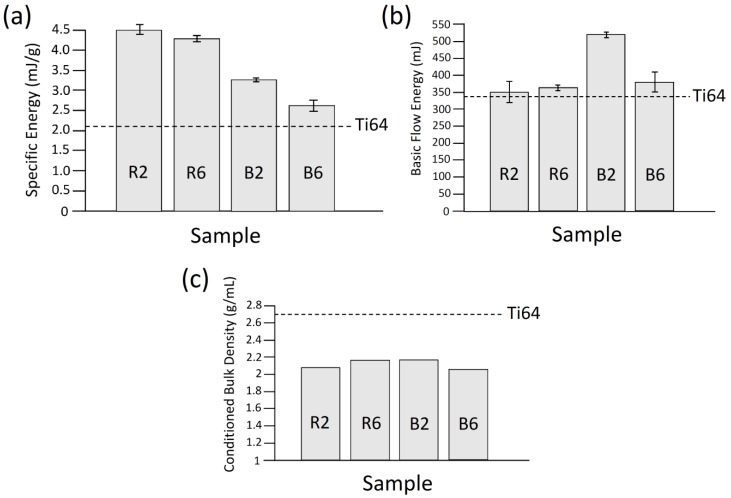
The flow behavior and conditioned bulk density results for 5 wt.% B_4_C/Ti-6Al-4V systems prepared by regular mixing and ball-milling methods with different mixing times: (**a**) specific energy (SE), (**b**) basic flow energy (BFE), and (**c**) conditioned bulk density (CBD) of the composite powder systems compared to the reference powder (Ti64).

As can be observed in Figure 9a,b, all of the developed composite powder systems showed lower flowability (higher BFE and SE) as compared to the host powder (Ti64). The flow response of the composite powders was found to be dependent on the blade movement direction. In downward movement (BFE), the ball-milled composite powders exhibited higher flow energy (lower flowability) compared to the regularly mixed composite systems (Figure 9b). However, considering the upward movement of the blade (SE), the ball-milled powders showed better flowability (Figure 9a). The major difference between the BFE and SE is the confined flow of the powder in the former case due to the effect of the bottom of the vessel. Since the recoater interacts with powder in an unconfined state during the powder layer deposition process, the SE is a better representative of the powder flow in PBF–AM processes.

As the microstructural and sphericity characterizations revealed in Figure 5, Figure 6 and Figure 8, the plastic deformation induced with longer milling times led to some degrees of shape change for the host powder particles from spherical to quasi-spherical/moderately flattened. In addition, the surface roughness of the host particles increased. The surface roughening (not the shape change) of Ti64 particles in the ball-milled composite powders subjected to relatively short mixing times (1–3 h) can be attributed mainly to the guest B_4_C particles hitting their surface due to two reasons:

- The guest B_4_C powder particles are noticeably harder than the host Ti64 powder particles [22,74,75,76]. Accordingly, the noticeably harder B_4_C particles have a great potential to scratch, punch and roughen the surface of softer Ti64 particles. Since they have the same hardness, the host–host inter-particle collisions might not affect the surface roughness of the host particles.
- As the microstructural observations of the starting powders revealed (Figure 3), the guest B_4_C particles have an irregular shape as opposed to the spherical shape of the host Ti64 particles. The collision of irregular-shaped B_4_C particles with the spherical-shape Ti64 particles has a higher chance of making the surface of Ti64 particles rough compared to the host–host collisions.

It is worth noting that the guest B_4_C particles affect the surface roughness of the host Ti64 particles only if the extreme guest–host collisions are provided by the metallic balls (ball milling). As the microstructural characterizations revealed, the regular mixing did not affect the surface roughness of Ti64 particles even at relatively long mixing times such as 6 h (Figure 4).

**Figure 10:**
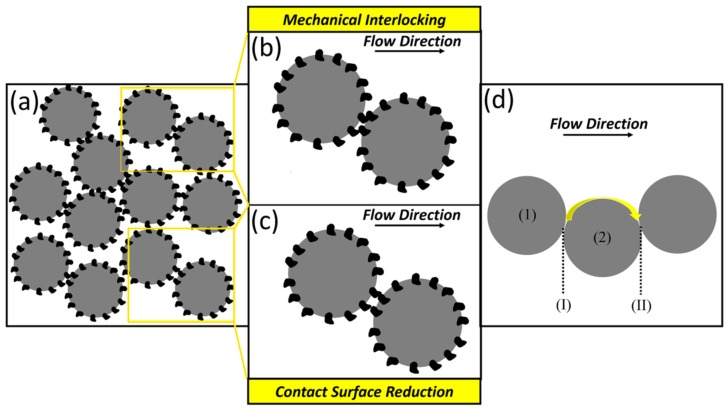
Schematic illustration presenting: (**a**) an overview of composite powder system with guest-decorated host particles; (**b**,**c**) close view of the selected regions showing “Mechanical Interlocking” and “Contact Surface Reduction” mechanisms, respectively; and (**d**) the contact area of two host powder particles when flowing relative to each other.

The increase in the SE (decreased flowability) in the B2 feedstock compared to the Ti64 reference sample may be attributed to the contribution of two different factors: (i) the change in the particle morphology [77], and (ii) the decoration of the host particles by the guest particles. The slight spherical to quasi-spherical shape change and the surface roughening of the host particles seem not to play major roles on the reduction of powder flowability of the B2 sample. Therefore, the considerable increase in the SE for this system could be related to the presence of the decorating guest powder particles. The corresponding suggested mechanism is schematically illustrated in Figure 10. The guest-decorated host powder particles may experience two different interactions during their flow. While the “mechanical interlocking” mechanism decreases the flowability by resisting the free flow of the powder particles relative to each other (Figure 10b), the “contact surface reduction” mechanism may improve the flowability of the system [78] (Figure 10c). Compared to the non-decorated host powder system, the inter-particle tangling caused by the decorating guest particles leads to the enhanced flow resistivity. On the other hand, if not forming tangles, the presence of the decorating guest particles lowers the contact surface area required for the movement of a guest-decorated host particle (particle 1) from position (I) to position (II) relative to another particle assumed to be fixed (particle 2) (as shown in Figure 10d). By reducing the inter-particle friction and adhesion force, this mechanism can improve the flowability [78,79,80]. As mentioned earlier, although the slight change in the morphology of the host powder particles in the B2 case could have adverse effects on the flowability, these factors seem not to be predominant in the flowability reduction due to their negligible deviation from the starting host powder particles. Accordingly, the significant decrease in the flowability of the B2 powder system could be due to the dominance of the “mechanical interlocking” mechanism over the “contact surface reduction” mechanism. The contribution of the “mechanical interlocking” and the “contact surface reduction” mechanisms in the overall powder flowability is a major function of the guest particle size. When nano-scale guest powder particles are deposited on the surface of the primarily cohesive host powder particles, the artificially generated nano-scale roughness has been reported to enhance the flowability [79,81,82,83]. Based on the mechanism provided in Figure 10, this could be attributed to the “contact surface reduction” combined with the lack of active “mechanical interlocking” sites in such composite powder systems, reducing the chance of particle entangling.

**Figure 11:**
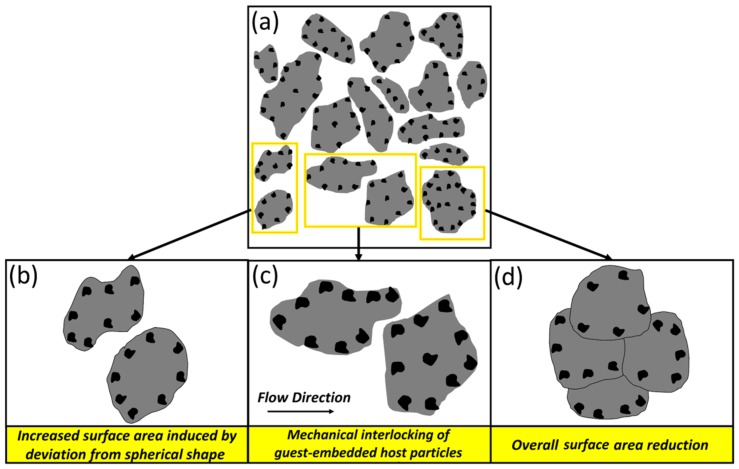
Schematic view presenting: (**a**) an overview of composite powder system with guest-embedded host particles; (**b**–**d**) close view of the selected regions showing “Increased Surface Area”, “Host Particles Mechanical Interlocking” and “Surface Area Reduction” mechanisms, respectively.

As indicated in Figure 9a, the B6 sample showed lower SE (better flowability) than the B2 one. In order to describe this finding, the mechanisms influencing the flowability of the B6 sample need to be explored. Referring to Figure 11, the flowability of the B6 powder feedstock is determined by the contribution of three mechanisms. Although the significant spherical to flattened/irregular shape change as well as the inter-locking of the guest-embedded host particles in such a system act to decrease the flowability [84,85,86], the increased particle size caused by the dominancy of the cold-welding over the fracture mechanism may reduce the effective surface area of the powder particles and, consequently, favor higher flowability [86] (Figure 7e and Table 2). Therefore, the overall flow behavior of such composite powder systems is governed by the competition among these mechanisms. The slight dominance of the inter-locking and shape change mechanisms over the surface area reduction in the B6 system is believed to be the reason for the small increment in its SE compared to the Ti64 case (Figure 11).

Referring to Figure 9a, the regularly mixed powder systems depicted SEs about twice that of the host particles. The observed phenomenon can be discussed based on the microstructural characterization of the powder systems. As shown in Figure 4 and Figure 8, the regularly mixed powder feedstocks had the guest particles almost non-attached to the completely spherical (non-deformed) host particles. Accordingly, neither the host particle shape change nor the decoration-induced tangling can be the reason behind the lower flowability of regularly mixed powders. However, the enhanced inter-particle friction caused by the presence of fine non-attached guest powder particles with an extremely high surface-to-volume ratio can explain the drastic increase in the SE of these samples.

#### 3.4.2. Conditioned Bulk Density (CBD)

The packing density of the powder as the starting material in the PBF–AM processes has a significant influence on the quality of the parts produced. The density of the powders in this study was analyzed by their conditioned bulk density (CBD). Referring to Figure 9c, regardless of the mixing method and time, incorporation of the guest powder particles to form composite powder feedstocks decreased the CBD compared to the starting host powder (Ti64). A portion of this decrease is due to the addition of a less-dense material (B_4_C) to the Ti64 powder. Moreover, the increased inter-particle friction arising from the irregular shape of the B_4_C particles combined with the large particle size distributions acts to reduce the packing density of the composite powder feedstocks compared to Ti64 powder [83,87,88]. In general, the higher random loose packing provided by the lower friction among the powder particles results in higher CBD. In the case of starting host powder particles, there is only the host/host inter-particle friction which determines the powder density. However, the introduction of the guest particles into the host powder leads to the emergence of new friction sources, namely, host/guest, and guest/guest inter-particle frictions which could be responsible for the lower CBD of the composite powders as compared to the Ti64 host powder.

Presence of free (non-attached) guest particles in the composite powder leads to the guest/host and guest/guest inter-particle frictions in addition to the host/host ones. According to Figure 9c, the higher CBD of the R6 sample compared to the R2 one is due to the lower amounts of free B_4_C particles existing in the system (Figure 4). Attachment of the guest to the host powder particles eliminates the host/guest inter-particle friction, which leads to the decrease in the host/host inter-particle friction by reducing their contact surface area. The morphology of powder particles also has a significant impact on the packing density of the powder bed and consequently, the density of the final component [89,90]. While the full decoration of the host particles by the guest particles in the B2 system promotes the attainment of a higher CBD, the slight deviation of the host particles from a spherical shape adversely influences the density (Figure 6b,c and Figure 8b). Therefore, the same CBD as that of the R6 system was obtained for the B2 case. The significant morphological change of the host particles in the B6 sample could be the main reason behind its low CBD. The flattened/irregular shape of the powder particles in the B6 system renders a poor packing due to the elevated inter-particle friction [87]. It is also worth noting that the formation of agglomerated guest particles in the regularly mixed composite systems adversely affects their potential in occupying the host particles interstices.

### 3.5. Material Loss

**Figure 12:**
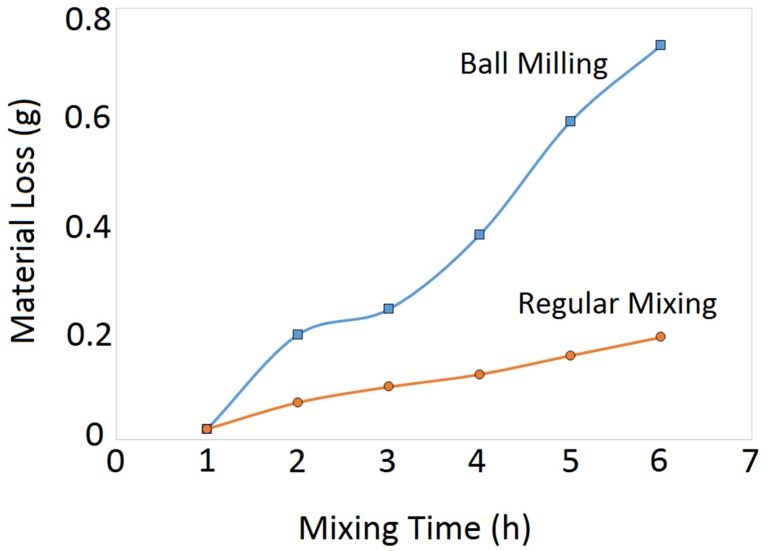
Material loss as a function of the mixing time for the regularly mixed and ball-milled 5 wt.% B_4_C/Ti-6Al-4V composite powders. The starting powder mixture was 100 g, and the ball-to-powder ratio was 5:1.

The mechanical mixing processes involves some material loss due to the cold-welding of the powder particles to the balls and/or jar. Therefore, the amount of the starting and final powder needs to be quantified. Figure 12 presents the variation in the powder mass for both the regularly mixed and ball-milled composite powders as a function of the mixing time. The material loss for both the regularly mixed and ball-milled cases is negligible (<1%). However, the regular mixing resulted in lower material loss than the ball-milling method due to the absence of balls.

### 3.6. Selection of the Best Possible Composite Powder

The ideal mixed powder system for the PBF–AM processing of MMCs needs to have: (i) non-free guest powder particles which are uniformly and homogeneously distributed throughout the system, (ii) host powder particles preserving their desired spherical shape, and (iii) the same flow behavior and apparent packing density as the starting host powder constituent. Although preserving the spherical shape of the host particles, the inadequate guest–host attachment associated with the regular mixing results in heterogeneous final MMC parts with improper distribution of the guest particles (or in-situ formed phases) or even their agglomeration. In addition, the regularly mixed composite powders showed noticeably lower flowability compared to the ball-milled composite systems. These issues restrict the implementation of the regularly mixed (R2 and R6) powder feedstocks in the PBF–AM of MMCs. Ball milling of the powders for relatively long milling times (B6) significantly improves the distribution state of the guest particles throughout the system by their embedment into the host powder particles. However, the significant spherical to flattened/irregular shape change and the particle coarsening are the main drawbacks of such powder feedstocks (Figure 7 and Figure 8 and Table 2). While the particle coarsening issue can be solved by sieving, this strategy may not be cost-and-time effective, especially for high-priced materials. When employing shorter milling times (2 h), the composite powder system showed almost spherical host particles fully decorated by the guest powder particles, meeting some of the requirements mentioned for the ideal composite powder feedstock (best possible case). Although such a powder ensures achieving homogenous MMC parts due to the proper host/guest attachment and the lack of non-attached (free) agglomerated guest particles, the decorating guest particles act as obstacles to the free flow of host particles and consequently, sacrifice the flowability. Proper selection of the recoater speed in PBF–AM processes could be a strategy to solve this issue when using such composite powder feedstocks [91]. Based on the above discussions, the composite powder system prepared by a relatively short milling time of 2 h (B2) is suggested as the best possible case for PBF–AM processes.

## 4. Conclusions

The characteristics of the 5 wt.% B_4_C(guest)/Ti-6Al-4V(host) mixed powder systems were studied from a powder bed fusion (PBF)–additive manufacturing (AM) perspective. For this purpose, the regular mixing and the ball-milling methods were employed with a wide range of milling times (1–6 h) to produce composite powder feedstocks. The developed powders were examined using microstructural characterization, phase formation, particle size, size distribution, sphericity, apparent density, and flow behavior analyses. Moreover, the mechanisms that play a role in the flowability of the mixed powder systems were analyzed based on the microstructural observations and the flow measurement results. The main outcomes can be outlined as follows:

1. With the regular mixing, the shape of the host powder particles remained unchanged until 6 h of mixing. The ball-milling method led to the change in the shape of host powder particles from spherical to quasi-spherical and then to a flattened/irregular shape by increasing the milling time, resulting in the decreased particle sphericity compared to the starting host particles.
2. The regular mixing method did not provide acceptable attachment of the guest B_4_C particles to the host particles. However, milling times as short as 2 h in the ball-milling case provided the host particles with a full decoration by the guest particles. Longer milling time (6 h) led to the guest particles embedded in the severely deformed host particles.
3. Although the basic flow energy (BFE) results contradict the specific energy (SE) measurements, the SE is believed to be a better representative of the powder layer deposition during PBF–AM process due to the unconfined and low-stress state of the powder.
4. Although being highly dependent on the mixing process variables, the flowability of the developed composite powders was lower than that of the reference Ti-6Al-4V powder. The regularly mixed and ball-milled composite powders exhibited ~110%, and 24–57% increase in SE compared to the Ti-6Al-4V powder, respectively.
5. The ball-milled feedstocks showed lower SE (better flowability) than the regularly mixed powders. The flow behavior of developed composite feedstocks was discussed based on the underlying mechanisms.
6. The produced composite powder systems showed 18–24% decrease in density compared to the reference Ti-6Al-4V powder.
7. The composite powder benefitting from fully decorated spherical-shape host particles is suggested as the best possible mechanically processed feedstock for PBF–AM processes. The relatively low flowability of this powder system should be considered when defining the recoater speed in PBF–AM processes.

## Figures and Tables

**Table 1:** Nominal chemical composition of the starting Ti-6Al-4V and B_4_C powders.

Powder	Elements (wt.%)								
**Ti-6Al-4V (Host)**	Ti	Al	V	Fe	O	N	C	H	B
	Bal.	6.3	4.0	0.03	0.1	0.01	0.01	<0.1	-
**B_4_C (Guest)**	B_4_C	Al	V	Fe	O	N	Free C	H	Free B
	Bal.	<0.001	-	<0.001	<0.04	<0.001	3	-	4

**Table 2:** The particle size distribution results presenting the cumulative distribution of D10, D50, and D90 for the Ti-6Al-4V (Ti64) and the developed composite powders.

Sample	D10	D50	D90
**Ti64**	23.39 ± 0.515	35.04 ± 0.360	45.71 ± 0.878
**R2**	18.81 ± 0.564	33.91 ± 0.420	45.24 ± 0.497
**R6**	20.12 ± 0.070	33.92 ± 0.310	45.49 ± 0.570
**B2**	22.89 ± 0.061	37.01 ± 0.362	51.28 ± 0.210
**B6**	25.20 ± 0.207	40.30 ± 0.221	61.94 ± 1.437
